# Supplementary material for: Does the human placenta express the canonical cell entry mediators for SARS-CoV-2?
Source: eLife. 2020 Jul 14;9:e58716. doi: 10.7554/eLife.58716 (PMC7367681; doi:10.7554/eLife.58716)
Supplement: Supplementary file 2. [file elife-58716-supp2.docx]

Supplementary File 2. Clinical and demographic characteristics of the study population from which placental samples were collected for snRNAseq studies

|  | **Study group**  **(n=32)** |
| --- | --- |
| **Clinical parameters** | |
| Maternal age (years; median [IQR]) | 25 (21.8-29) |
| Body mass index (kg/m^2^; median [IQR]) | 27.8 (21.7-31.6)^a^ |
| Primiparity | 15.6% (5/32) |
| Cesarean section | 59.4% (19/32) |
| Gestational age at delivery (weeks; median [IQR]) | 36.9 (32.9-39.1) |
| Birthweight (g; median [IQR]) | 2802.5 (1833.8-3233.8) |
| **Ethnicity** | |
| African-American | 81.3% (26/32) |
| Caucasian | 6.2% (2/32) |
| Other | 12.5% (4/32) |

Data are given as median (interquartile range, IQR) and percentage (n/N)

^a^ One missed data
