# Supplementary material for: Does the human placenta express the canonical cell entry mediators for SARS-CoV-2?
Source: eLife. 2020 Jul 14;9:e58716. doi: 10.7554/eLife.58716 (PMC7367681; doi:10.7554/eLife.58716)
Supplement: Supplementary file 3. [file elife-58716-supp3.docx]

**Supplementary File 3. Bulk Gene Expression Data Analysis of ACE2 and TMPRSS2 in the placental tissues**

| **Study** | **Affymetrix probeset** | **Symbol** | **Log_2_ Average Expression** | **% of samples detected above background** |
| --- | --- | --- | --- | --- |
| Kim et al. | 219962_at | ACE2 | 4.61 | 90% |
| Kim et al. | 222257_s_at | ACE2 | 5.71 | 70% |
| Kim et al. | 205102_at | TMPRSS2 | 5.97 | 10% |
| Kim et al. | 1570433_at | TMPRSS2 | 3.37 | 0% |
| Kim et al. | 211689_s_at | TMPRSS2 | 2.96 | 0% |
| Kim et al. | 226553_at | TMPRSS2 | 6.02 | 0% |
| Toft et al. | 219962_at | ACE2 | 4.26 | 82% |
| Toft et al. | 222257_s_at | ACE2 | 5.18 | 57% |
| Toft et al. | 1570433_at | TMPRSS2 | 3.30 | 0% |
| Toft et al. | 205102_at | TMPRSS2 | 5.78 | 0% |
| Toft et al. | 211689_s_at | TMPRSS2 | 3.34 | 0% |
| Toft et al. | 226553_at | TMPRSS2 | 5.65 | 0% |
